# Vitiligo and chronic autoimmune thyroiditis

**DOI:** 10.25122/jml-2019-0134

**Published:** 2021

**Authors:** Florica Sandru, Mara Carsote, Simona Elena Albu, Mihai Cristian Dumitrascu, Ana Valea

**Affiliations:** 1. Department of Dermatology, Elias Emergency University Hospital, Bucharest, Romania; 2. Department of Dermatology, Carol Davila University of Medicine and Pharmacy, Bucharest, Romania; 3. Department of Endocrinology, C. I. Parhon National Institute of Endocrinology, Bucharest, Romania; 4. Department of Endocrinology, Carol Davila University of Medicine and Pharmacy, Bucharest, Romania; 5. Department of Obstetrics and Gynecology, Emergency University Hospital, Bucharest, Romania; 6. Department of Obstetrics and Gynecology, Carol Davila University of Medicine and Pharmacy, Bucharest, Romania; 7. Department of Endocrinology, Clinical County Hospital, Cluj-Napoca, Romania; 8. Department of Endocrinology, Iuliu Hatieganu University of Medicine and Pharmacy, Cluj-Napoca, Romania

**Keywords:** vitiligo, thyroiditis, antibodies, ATG – anti-thyroglobulin antibodies, FOXD3 – forkhead transcription factor D3, IFN – interferon, IL – interleukin, JAK-STAT – Janus kinase-signal transducer and activator of transcription, ROS – reactive oxygen species, TGF – transforming growth factor, TPO – thyroid peroxidase antibodies

## Abstract

Vitiligo, the discoloration of the skin, has different autoimmune mechanisms reflected by many biomarkers as shown by skin histology, staining for CD4 and CD8 T lymphocytes, chemokine ligand 9 or circulating cytokines such as interleukin (IL)-1 beta, interferon (IFN)-gamma, transforming growth factor (TGF)-beta, antibodies, markers of oxidative stress, chemokines, and others. In this narrative review, we aim to overview vitiligo in relationship with chronic autoimmune thyroiditis. Regarding vitiligo, more than 50 different genetic loci have been associated with this disease, and the heritability is high. There is a 20% risk of an environmental connection which may also act as a trigger; moreover, the association with human leukocyte antigen (HLA) expression is well recognized. The specific lesions display CD8+ tissue-resident memory T cells as continuous key activators of melanocytes. The association with chronic thyroiditis is based on common autoimmune background and excessive reactive oxygen species that destroy melanocytes and thyrocytes (oxidative stress hypothesis) with thyroxine and melanin as target molecules, thus sharing a common origin: tyrosine. Moreover, common epigenetic anomalies or mutations of the Forkhead transcription factor D3 (FOXD3) have been described. Since vitiligo affects up to 1–2% of the population worldwide and 34% of patients have positive thyroid antibodies, apart from common autoimmunity background and oxidative stress toxicity, the association is clinically relevant for different practitioners.

## Introduction

Vitiligo represents the discoloration of the skin, a condition with rather limited therapeutical options nowadays [1]. Regarding the etiology, the autoimmune component is well known, but the exact mechanisms are still a matter of debate [2, 3]. The mechanisms of this condition are reflected by biomarkers like skin histology, including additional staining for CD4 and CD8 T lymphocytes on the one hand, or markers like chemokine ligand 9, different circulating cytokines: interleukin (IL)-1 beta, interferon (IFN)-gamma, transforming growth factor (TGF)-beta, antibodies, markers of oxidative stress, chemokines on the other hand [2, 3].

Another example is IL-17, which is connected with different autoimmunity conditions, and its level is high in vitiligo; in addition to the lesion’s clinical aspects, a positive correlation with disease activity has been described [2, 3]. Moreover, IL-17 may be modulated by ultraviolet B phototherapy, which is used for vitiligo [2, 3]. IL-17 has been linked with other chronic skin inflammation like psoriasis, acne, atopic dermatitis, and autoimmune-derived diseases like alopecia areata [4]. Poor prognosis factors are clinically described as Koebner’s phenomenon, confetti-like lesions, and poorly defined borders [5].

Since vitiligo impacts the quality of life, different therapies are under consideration, and phototherapy remains the first-line approach which represents an option for different dermatoses as well [6–8]. Early recognition and prompt therapy seem to improve the prognosis [9]. The efficacy of other alternatives like platelet-rich plasma is still insufficiently documented [10]. In this paper, we aimed to overview vitiligo in relationship with chronic autoimmune Hashimoto’s thyroiditis.

## Material and Methods

We conducted a narrative review based mainly but not exclusively on PubMed database research. A number of 50 references were found and are described in detail in this paper.

## Results

### Vitiligo

The melanocytes’ progressive autoimmune deterioration causes lesions at the level of skin and hair [11]. The process is linked to both genetic and environmental factors, and multiple aspects need to be taken into consideration when it comes to discussing the complex pathogeny of the condition.

More than 50 genes carry the disease susceptibility. The heritability percent is increased, and there is a 20% risk of an environmental connection that may also act as a trigger in some cases. [11] The association with human leukocyte antigen (HLA) expression is well recognized [11]. Immune anomalies are underlining vitiligo and also connecting it with the risk of developing melanoma [12]. Signal transduction pathways like the Janus kinase-signal transducer and activator of transcription (JAK-STAT) play a role in the pathogenic loop of vitiligo [13]. Drugs targeting the JAK-STAT pathway are encouraging, seem safe, and efficient in re-pigmentation but still irrelevant in daily practice on a larger scale [14, 15].

Apart from non-receptor tyrosine kinases pathways like JAK-STAT, signals from the tyrosine kinases network also seem to contribute to the autoimmune-related background involving the skin acquired condition [16]. Also, the lesions of vitiligo display CD8+ resident memory T cells as continuous key activators of the destruction of pigment-producing cells [17]. Another mechanism is related to the excessive local production of reactive oxygen species (ROS) in melanocytes about to be destroyed [18].

### Chronic autoimmune thyroiditis

The autoimmune Hashimoto’s thyroiditis has a relatively high frequency in the general population with a female predisposition and potential positive family history of autoimmune conditions [19, 20]. It is regarded as the most common autoimmune disease and the most important cause of hypothyroidism in iodine non-deficient areas. It is also the most common cause of hypothyroidism in children and teenagers nowadays [19, 20]. The clinical presentation may be irrelevant or autoimmune hypothyroidism (including the severe pattern of myxedema) may be detected [19, 20]. The presence of other endocrine and non-endocrine conditions may actually be the reason for admission [19, 20]. The thyroid gland could have a normal volume or be enlarged, typically without nodules [19, 20]. It underlines specific hypoechoic ultrasound features [19, 20]. Atrophic autoimmune myxedema has also been described, especially in the elderly, who usually have a reduced gland diameter [19, 20].

Thyroid antibodies that are causing the condition are well known and easily tested in everyday practice: thyroid peroxidase antibodies (TPO) and anti-thyroglobulin antibodies (ATG) [21, 22]. Increased antibody levels seen a least once on a blood test is enough to establish the diagnosis in daily practice [21, 22]. Their levels may be fluctuating in different patterns. Nonetheless, the most relevant for routine assessments are TPO [21, 22]. Genetic background causing the autoimmunity activation together with environmental factors including nutrients (like selenium deficiency) have been described as parts of the pathogen network [22–24].

Thyroid function may be normal in chronic autoimmune thyroiditis, but only for a while; in time, subclinical/clinical hypothyroidism develops due to the blocking effect of the antibodies on the thyroid [24, 25]. Transitory thyrotoxicosis can be rarely found [24, 25]. The pathological report shows lymphocytic infiltration of the thyroid and follicular cells may become atrophic [26]. Thyroid ultrasound remains the most helpful tool for diagnosis, except for antibody values [27]. Low thyroid function requires levothyroxine substitution, while thyroidectomy is rarely indicated unless local compressive symptoms are present [28].

However, 20% of patients associate at least one more autoimmune disease [29–31]. Regarding the malignancy risk, the condition has been correlated with papillary thyroid cancer, increasing the risk of primary thyroid lymphoma [29–31]. Due to the high prevalence in the general population, the overlap with other frequently found conditions like obesity, metabolic syndrome, or breast cancer has been analyzed, and the association is still a matter of debate [32–34].

### Vitiligo and chronic autoimmune thyroiditis

Vitiligo and Hashimoto’s thyroiditis represent two well-known autoimmune diseases, noting that vitiligo is the most common condition which involves skin depigmentation, found in up to 1–2% of the population [35]. The autoimmune background is common between the diseases; thus, the association involves more than antibodies [35]. Because of the incidental co-presence of the two conditions, thyroid antibodies are encouraged to be routinely tested in patients with vitiligo [36]. On the other hand, vitiligo is part of the non-endocrine conditions included in poly-glandular autoimmune syndrome as well as gastritis and hepatitis of autoimmune cause, celiac disease, pernicious anemia, and others [37]. The reported prevalence of positive thyroid antibodies is 34%, with different results depending on ethnicity [38, 39].

Another common hypothesis of the two disorders includes reactive oxygen species in high amounts that trigger conditions, destroying melanocytes and thyrocytes (oxidative stress hypothesis) [40, 41]. The target molecules thyroxine and melanin share a common origin: tyrosine [40,41]. A new therapeutic approach is under consideration using quinone derivatives [40]. Their role is to combat oxidative stress-related autoimmunity [40].

Also, common epigenetic anomalies have been previously described [42]. Mutations of the Forkhead transcription factor D3 (FOXD3) are linked to positive thyroid antibodies and the diagnosis of vitiligo [43]. Clinical presentation varies; one prevalence study identified positive antibodies in patients with vitiligo but with an asymptomatic thyroid condition [44]. The association is also found in young patients [45].

## Discussion

When it comes to vitiligo and non-thyroid autoimmune conditions, some studies pointed to a potential common association with diabetes mellitus. [46]. This may be or not a part of a polyglandular autoimmune syndrome. We should also mention premature ovarian failure (the ovarian function stops before the age of 40), which includes the fact that one-third of females have autoimmune morbidities, including vitiligo and Hashimoto’s thyroiditis [47].

The most frequent is chronic thyroiditis, but vitiligo has also been described with a higher risk than the general population [47]. A sex-chromosomal disorder that increases the risk of any autoimmune disease is Turner syndrome [48, 49]. The syndrome includes a higher prevalence of autoimmune conditions, which are seen twice as much compared to normal women, and these disorders involve vitiligo, chronic thyroiditis, type 1 diabetes mellitus, celiac disease, and alopecia areata [48, 49]. In this particular situation, the contributor to increased autoimmunity is the iXq chromosome and exposure to estradiol (due to iatrogenic complications) [48, 49]. Another genetic disease with a higher risk for both conditions, vitiligo and Hashimoto’s thyroiditis, is Down syndrome, potentially with a more severe phenotype for the thyroid condition [50].

## Conclusion

Since vitiligo affects up to 1–2% of the population worldwide and 34% of them have positive thyroid antibodies, apart from common autoimmunity background and oxidative stress toxicity, this association is clinically relevant for practitioners.

## Acknowledgments

### Conflict of interest

The authors declare that there is no conflict of interest.
